# Structure–Activity Relationships of Natural and Synthetic Anthraquinones: Relevance for Pharmacology and Nutritional Supplementation

**DOI:** 10.3390/ijms27208960

**Published:** 2026-10-09

**Authors:** Nataliya V. Yaglova, Sergey S. Obernikhin, Elina S. Tsomartova, Elizaveta V. Chereshneva, Dibakhan A. Tsomartova

**Affiliations:** 1 Laboratory of Metabolomic and Proteomic Analysis, Federal Research Centre of Nutrition, Biotechnology and Food Safety, 109240 Moscow, Russia; ober@mail.ru; 2 Department of Human Anatomy and Histology, Federal State Funded Educational Institution of Higher Education I.M. Sechenov First Moscow State Medical University, 119435 Moscow, Russia; tselso@yandex.ru (E.S.T.); yelizaveta.new@mail.ru (E.V.C.); dtsomartova@mail.ru (D.A.T.)

**Keywords:** anthraquinones, structure–activity relationship, dietary supplements, drug design, pharmacological effects

## Abstract

Anthraquinones constitute an extensive class of natural and synthetic derivatives. Natural anthraquinones are widely utilized as active ingredients in dietary supplements and traditional medicine. However, their inherent polypharmacology frequently complicates their biomedical application; while a broad spectrum of biological activities is advantageous in defined clinical settings, it often triggers off-target or adverse reactions under other conditions. Consequently, standardizing both chemical composition and biological efficacy remains a critical challenge for drug development and supplement formulation. Although extensive research has focused on the chemistry and pharmacology of anthraquinones, the underlying structure–activity relationships (SARs) remain fragmented across individual studies, lacking a unified framework that correlates substituent effects and electronic properties with specific biological endpoints. This review systematically evaluates the relationship between the chemical structure of anthraquinones and their biological properties. Specifically, we analyze how the pharmacological profiles and toxicity of anthraquinones are governed by three levels of structural modification: simple monomers, dimeric systems, and hybrid architectures, ultimately providing structure-guided principles for designing optimized therapeutics and safer dietary supplements.

## 1. Introduction

Anthraquinones constitute a large and structurally diverse group of natural and synthetic compounds that find use both as medicinal products and as dietary supplements. In the clinic, anthraquinone derivatives are prescribed for specific indications, most prominently as laxatives and, in selected cases, as anti-inflammatory or antineoplastic agents. Standardized senna and cascara preparations exemplify the anthranoid-based medicinal products, while synthetic anthraquinone derivatives doxorubicin, daunorubicin, epirubicin, and mitoxantrone, occupy a well-established place in oncology [1,2,3]. Dietary supplements, by contrast, rely on anthraquinone-producing plants or their extracts, among them senna, aloe, cascara, and rhubarb. These products are marketed for bowel regulation, weight management, antioxidant support, or general wellness, and their long-standing use in traditional medicine contributes to a perception of safety among consumers.

The two product categories are nevertheless not interchangeable. Unlike medicines, dietary supplements may be purchased and consumed without medical supervision, and they can differ substantially in the identity and concentration of their active constituents. Classifying a product as a supplement does not remove the need for quality control or safety evaluation, especially when the product contains concentrated botanical extracts or is intended for prolonged use. For anthraquinone-containing supplements in particular, standardization of active constituents, accurate labeling, batch-to-batch consistency, appropriate dose limits, and clear warnings against extended unsupervised intake all take on particular importance.

Standardizing composition and effect remain difficult. One promising route toward safer anthraquinone-containing supplements is the substitution of poorly defined herbal components with single substances whose effects and toxicological parameters have been characterized. The anthraquinone scaffold lends itself unusually well to structure–activity relationship analysis because its biological activity is governed by a limited number of physicochemical parameters that can, to a large extent, be tuned independently. The electron affinity of the quinone moiety determines the propensity for one-electron reduction and the generation of reactive oxygen species (ROS) [4,5,6,7]. The presence and spatial arrangement of peri-hydroxyl groups dictate the capacity for metal chelation, which can either suppress Fenton chemistry (thereby lowering toxicity) or enable selective metalloprotein inhibition [8,9,10,11]. The planarity and π-electron surface of the tricyclic core control the geometry and affinity of DNA intercalation [12,13]. Finally, lipophilic substituents—methyl, methoxy, or glycosidic groups—regulate membrane permeability, bioavailability, and subcellular distribution [14,15,16].

These parameters are not independent, and this is precisely where the design problem becomes interesting. A hydroxyl group at C-1 simultaneously raises the electron affinity of the quinone (favoring semiquinone formation), provides a chelation site for Fe^2+^ or Cu^2+^, and supplies a hydrogen-bond donor that stabilizes DNA intercalation [8,9,17]. Conversely, O-methylation or glycosylation of the same hydroxyl removes both redox activity and metal binding while markedly changing pharmacokinetic behavior [18,19]. Enhancing one activity, in other words, often amplifies an unwanted liability.

Recent work in anthraquinone medicinal chemistry has moved well beyond simple monomers. Dimeric structures such as hypericin show enhanced photodynamic activity [20,21,22,23,24,25,26,27,28], and hybrid molecules that covalently link the anthraquinone core to heterocyclic pharmacophores (triazoles, furans, chalcones) or peptide motifs allow multitarget modulation and can overcome drug resistance [29,30,31,32,33,34,35,36,37]. The SAR principles that govern these more complex architectures, however, remain fragmented across studies, and no comprehensive framework yet integrates substituent effects, electronic mechanisms, and biological endpoints and provides an integrated cross-class SAR framework that spans monomeric, dimeric, and hybrid architectures within a unified chemical–mechanistic–biological logic. This review analyzes systematically how the chemical structure of anthraquinones, from monomeric derivatives through dimeric to hybrid architectures, determines their pharmacological profiles. In the final sections we examine the toxicity and adverse effects of the anthraquinones and outline rational strategies for reducing side effects while retaining activity.

Scope and literature basis. This work is a structured narrative review organized around three levels of structural complexity (monomeric, dimeric, and hybrid anthraquinones). Literature was retrieved from PubMed, Scopus and Web of Science using combinations of the terms “anthraquinone”, “structure–activity relationship”, “redox potential”, “metal chelation”, “DNA intercalation”, “dietary supplement” and the names of individual compounds, with emphasis on primary studies published between 2000 and 2026 and seminal earlier work where mechanistically relevant. Priority was given to reports providing experimental physicochemical or biological data. Because this is not a systematic review, no formal inclusion/exclusion scoring was applied; selection was based on relevance to the structure–activity theme and, where possible, on availability of quantitative data.

## 2. Anthraquinone Scaffold as the SAR Platform

### 2.1. Structural Features of the Anthraquinone Core

The anthraquinone core consists of a tricyclic anthracene backbone bearing carbonyl groups at positions 9 and 10, giving the formal structure anthracene-9,10-dione (Figure 1A). This quinoid system presents an extended, planar aromatic framework roughly 7.2 Å along its long axis, 4.6 Å across the short axis, and about 3.4 Å thick [12]. The unsubstituted molecule possesses C_2_ᵥ symmetry, with the C-9 and C-10 carbonyl groups lying in the plane of the tricyclic aromatic system, their C=O bonds conjugated with the ring π-system and directed outward along the short molecular axis. This coplanar, fully conjugated arrangement is what gives the scaffold its planar, quinoid electronic character.

Substitution positions are numbered 1–8 on the peripheral aromatic rings. Positions 1, 4, 5, and 8 are designated α (peri to the quinone carbonyls); positions 2, 3, 6, and 7 are designated β. This positional nomenclature has chemical consequences. An α-hydroxyl group, for instance at C-1 or C-8, can engage in intramolecular hydrogen bonding with the adjacent carbonyl, forming a six-membered pseudochelate ring (Figure 1B) [8,9]. Density functional theory (DFT) calculations estimate the strength of this hydrogen bond at 8–12 kcal/mol, unusually strong by organic standards, and it confers enhanced acidity on the phenolic proton: pK_a_ ≈ 6.5–7.5 for 1-hydroxyanthraquinone compared with ≈10 for simple phenols [8]. β-Hydroxyl groups lack this stabilizing interaction and exhibit pK_a_ values typical of unactivated phenols (≈9.5–10.5). The first phenolic pKa values of polyhydroxyanthraquinones typically fall in the range of approximately 7–11 (experimentally determined) but depend strongly on the number, position, and electron-withdrawing or electron-donating character of substituents, as well as on solvent and ionic strength; computed values for less-studied derivatives should be treated as estimates.

The quinone carbonyls are electron-withdrawing and render the aromatic rings electron-deficient. Calculated Mulliken atomic charges place a partial negative charge (δ ≈ −0.50 to −0.55 e) on the carbonyl oxygens and a partial positive charge (δ ≈ +0.40 e) on the C9 and C10 carbons, making them electrophilic [8]. Electron-donating substituents such as –OH or –NH_2_ at α positions partially offset this electron depletion through resonance, raising the HOMO energy and lowering the reduction potential. Electron-withdrawing groups (–COOH, –Cl, –NO_2_), by contrast, further deplete electron density, making the quinone easier to reduce (Figure 1C) [17,38].

Natural anthraquinones fall into two structural types based on the distribution of hydroxyl groups [39]. The alizarin type clusters hydroxyls on a single aromatic ring (alizarin itself carries 1,2-dihydroxy; purpurin, 1,2,4-trihydroxy). The rhein type distributes hydroxyls across both rings (emodin, 1,3,8-trihydroxy; chrysophanol and rhein, both 1,8-dihydroxy, rhein additionally carrying a carboxyl at C-3). This classification has pharmacological relevance. Alizarin-type compounds, possessing an ortho-dihydroxyl (catechol) motif, show superior antioxidant activity because the catechol stabilizes the semiquinone radical through extended conjugation [19,20,23]. Rhein-type compounds lack the catechol and display weaker radical-scavenging ability, but they possess superior antibacterial and laxative activities, attributed to higher membrane permeability and interaction with bacterial enzymes [28,29,30,31,32].

### 2.2. Physicochemical Consequences of Planarity and Conjugation

The rigid planarity of the scaffold imposes strict geometric constraints that determine how anthraquinones interact with biological macromolecules. DNA intercalation, one of the primary mechanisms behind antitumor activity, requires that the planar aromatic surface slips between stacked DNA base pairs, which are separated by roughly 3.4 Å [12,13]. The anthraquinone core, with a thickness of ≈3.4 Å, satisfies this requirement precisely. Intercalation induces local unwinding of the DNA helix (typically 18–26° per intercalator) and lengthens the duplex by ≈3.4 Å per inserted molecule. This distortion inhibits topoisomerases I and II, the enzymes that resolve topological strain during DNA replication and transcription [12,13,35,36,37].

Planarity also dictates how accessible substituents are to enzyme active sites. Anthra[2,3-b]furan-3-carboxamides, fused tricyclic derivatives with extended planar surfaces, show enhanced topoisomerase I inhibition (IC_50_ values in the nanomolar range) compared with simpler anthraquinones, because the expanded aromatic surface increases the contact interface with the DNA–enzyme complex [35,36,37]. Increased planarity, however, reduces aqueous solubility: unsubstituted anthraquinone dissolves at only ≈0.6 μg/mL at 25 °C, which limits bioavailability. Introduction of polar substituents (–OH, –COOH, –NH_2_) or glycosylation markedly improves solubility but usually at the expense of membrane permeability, a classic lipophilicity–solubility trade-off [14,15,18,19]. Poor aqueous solubility limits the dissolution rate and gastrointestinal concentration available for absorption, but overall oral bioavailability is determined by the combined contributions of solubility, intestinal permeability, presystemic gut-wall and hepatic metabolism, efflux transporter activity, and systemic clearance. Anthraquinones present challenges at multiple of these steps simultaneously, as discussed in Section 8.

The extended π-conjugation of the scaffold enables strong absorption in the UV and visible ranges. The lowest-energy electronic transition (HOMO→LUMO, π→π*) occurs at λ_max_ ≈ 320–340 nm for simple anthraquinones and shifts bathochromically to 400–600 nm in heavily hydroxylated or conjugated systems; hypericin, for example, absorbs at λ_max_ ≈ 590 nm [23]. This absorption underlies photodynamic activity. Upon light excitation, the molecule undergoes intersystem crossing to a long-lived triplet state (^3^AQ*), which can transfer energy to molecular oxygen (^3^O_2_), generating singlet oxygen (^1^O_2_) and superoxide radical (O_2_•^−^) [23,24]. The quantum yield of singlet oxygen generation (Φ_Δ_) is structure-dependent. Hypericin achieves Φ_Δ_ ≈ 0.73, among the highest for any organic photosensitizer, while simple anthraquinones yield Φ_Δ_ < 0.1 [23]. This large difference reflects the role of extended conjugation and heavy-atom effects (for instance, the presence of sulfur or nitrogen heteroatoms in hybrid structures) in promoting intersystem crossing [40,41,42]. Enhanced intersystem crossing in hybrid anthraquinone structures is most reliably produced by the incorporation of heavy atoms such as bromine or iodine, which increase spin–orbit coupling and thereby facilitate singlet-to-triplet transition. Sulfur and nitrogen heteroatoms can influence photophysical properties through other mechanisms like altered conjugation, charge-transfer character, and triplet energy, but do not intrinsically produce a heavy-atom effect equivalent to halogenation and should not be categorized as such.

Crystallinity, a direct consequence of planarity and symmetry, also affects pharmaceutical properties. Anthraquinone derivatives form highly stable crystal lattices held together by π–π stacking and hydrogen bonding, resulting in high melting points (alizarin, m.p. 289–290 °C; emodin, m.p. 255–257 °C) and low dissolution rates [43]. Poor dissolution is a recognized bottleneck for oral bioavailability and has driven formulation strategies including nanocrystal suspensions, liposomal encapsulation, and prodrug derivatization [44,45,46,47,48,49].

### 2.3. Redox Cycling, Chelation, and DNA Interaction

Anthraquinones exert biological activity through three principal, often overlapping mechanisms: redox cycling, metal chelation, and DNA intercalation (Figure 2).

#### 2.3.1. Redox Cycling and ROS Generation

One-electron reduction, catalyzed primarily by NADPH:cytochrome P450 reductase and related flavoproteins, generates the semiquinone radical anion (AQ•^−^), which re-oxidizes under aerobic conditions to regenerate the quinone and produce superoxide, the basis of ROS-generating pro-oxidant activity. Two-electron reduction, mediated by NQO1 (NAD(P)H:quinone oxidoreductase 1), bypasses the semiquinone intermediate and delivers the hydroquinone directly; depending on cellular context, this pathway may be cytoprotective rather than pro-oxidant. The two pathways are not interchangeable, and the biological outcome depends on which predominates, a function of reductase expression levels, oxygen tension, and intracellular glutathione status [4,5,6,7,50]. The semiquinone can either be back-oxidized by molecular oxygen, regenerating the parent quinone and producing superoxide (O_2_•^−^), or undergo a second one-electron reduction to the fully reduced hydroquinone (AQH_2_) [4,5,6,7]. The one-electron reduction potential (E_1_/_2_) of anthraquinones ranges from approximately −0.45 V to −0.80 V versus the saturated calomel electrode (SCE), depending on substitution [9,10] (Table 1). Because the parameters presented in Table 1 for the antioxidant activity of anthraquinones derive from heterogeneous experimental settings, we use them to illustrate qualitative structure–activity trends rather than to rank compounds numerically.

By convention, a less negative (more positive) one-electron reduction potential corresponds to a quinone that is more easily reduced. Reported values must therefore be interpreted together with the mechanism of radical stabilisation. α-Hydroxyl groups stabilise the semiquinone radical anion through intramolecular hydrogen bonding and proton-coupled electron transfer (PCET); this both facilitates single-electron acceptance from free radicals (hence the strong DPPH/ABTS (2,2′-azino-bis(3-ethylbenzothiazoline-6-sulfonic acid) scavenging of purpurin and alizarin) and lowers the kinetic barrier to reduction. The apparent paradox—that good scavengers can also be efficient redox cyclers—is resolved by context: under exogenous oxidative stress these compounds act as chain-breaking antioxidants, whereas in the presence of active cellular reductases the same low-barrier reduction drives semiquinone formation and superoxide generation (pro-oxidant behaviour). Purpurin (1,2,4-trihydroxy) exhibits E_1_/_2_ ≈ −0.45 V, whereas chrysophanol (1,8-dihydroxy) exhibits E_1_/_2_ ≈ −0.78 V [9,10]. This difference reflects stabilization of the semiquinone radical by intramolecular hydrogen bonding: the α-hydroxyl proton is labilized by the adjacent carbonyl, enabling proton-coupled electron transfer (PCET) that lowers the thermodynamic barrier to reduction [9,10]. Methoxy or acetoxy groups, which lack a mobile proton, cannot stabilize the radical in this way, and their reduction potentials remain high (more difficult to reduce) [10].

The biological consequence of facile one-electron reduction is intracellular ROS generation. Superoxide dismutase converts O_2_•^−^ to hydrogen peroxide (H_2_O_2_), which in the presence of free Fe^2+^ or Cu^2+^ can undergo Fenton chemistry to yield hydroxyl radical (•OH), the most reactive and damaging ROS [4,5,6,7,10,11]. This ROS burst causes lipid peroxidation, protein oxidation, and DNA strand breaks, which in turn trigger apoptosis [4,5,6,7,50,55,56,57,58,59]. The same ROS generation, however, underlies off-target toxicity. Cardiomyocytes express high levels of NADPH oxidases and possess limited antioxidant capacity, making them particularly vulnerable to quinone-induced oxidative damage—an observation that explains the cardiotoxicity of doxorubicin [60,61,62].

The antioxidant activity and redox toxicity of anthraquinones are, in this sense, two sides of the same coin. Compounds with low E_1_/_2_ (easy to reduce, such as purpurin and alizarin) act as efficient radical scavengers under conditions of exogenous oxidative stress, because they preferentially accept electrons from free radicals [8,9,53]. Under basal cellular conditions with active reductases, however, these same compounds cycle redox and generate ROS, exhibiting pro-oxidant behavior [50,63,64]. Summary parameters of antioxidant activity are presented in Table 1.

#### 2.3.2. Metal Chelation

Anthraquinones bearing hydroxyl groups at positions capable of forming five- or six-membered chelate rings (for example, 1,2-dihydroxy; 1,8-dihydroxy; 1-hydroxy-9-carbonyl) function as metal chelators [8,9,65,66,67,68,69,70] (Figure 2). The pharmacologically most relevant metal ions are Fe^2+^, Fe^3+^, Cu^2+^, Zn^2+^, and Mg^2+^, which serve as cofactors in metalloproteins including prolyl-4-hydroxylase (Fe^2+^), matrix metalloproteinases (Zn^2+^), tyrosinase (Cu^2+^), and topoisomerases (Mg^2+^) [65,68,69,70].

Alizarin (1,2-dihydroxyanthraquinone) is the prototypical chelator, forming stable 1:1 or 2:1 (ligand:metal) complexes with Fe^3+^ and Al^3+^ (log *K* ≈ 12–14 for Fe^3+^) [8,9]. The 1,2-dihydroxyl (catechol) motif is particularly effective because both oxygens lie in the plane of the aromatic ring, enabling coplanar coordination. By contrast, 1,8-dihydroxyanthraquinones such as chrysophanol and emodin chelate less effectively (log *K* ≈ 8–10 for Fe^3+^) because the hydroxyls lie on opposite sides of the tricyclic plane, forcing the metal into a distorted, out-of-plane geometry [8,9].

Chelation can modulate biological activity in two opposing ways. First, sequestration of catalytic metal ions inactivates metalloproteins. For instance, 2,7,8-trihydroxyanthraquinone inhibits prolyl-4-hydroxylase (IC_50_ ≈ 2 μM) by chelating the Fe^2+^ ion at the active site, which prevents substrate hydroxylation [65]. Similarly, anthraquinones inhibit matrix metalloproteinases (MMPs) by coordinating the catalytic Zn^2+^, which blocks extracellular matrix degradation and tumor invasion [68,69,70]. Second, chelation can suppress Fenton chemistry by sequestering free (non-protein-bound) Fe^2+^ or Cu^2+^, thereby preventing ROS generation and reducing oxidative toxicity [10,11]. This dual role, enzyme inhibition versus antioxidant protection, depends on whether the chelated metal is protein-bound (inhibitory effect) or free (protective effect).

Recent work indicates that anthraquinone–metal complexes can exhibit differential cytotoxicity toward cancer versus normal cells [10,11]. Fe^3+^ complexes of novel anthraquinone derivatives selectively inhibited viability of A549 lung cancer cells (IC_50_ ≈ 8 μM) while sparing MRC-5 normal lung fibroblasts (IC_50_ > 50 μM), whereas Cu^2+^ complexes showed no selectivity [10]. This suggests that dysregulated iron homeostasis in cancer cells, characterized by upregulated transferrin receptor and downregulated ferritin, renders them preferentially vulnerable to iron–chelator complexes [10,11]. The mechanistic basis for this selectivity, however, remains incompletely understood and warrants further investigation.

#### 2.3.3. DNA Intercalation

DNA intercalation by anthraquinones is driven primarily by π–π stacking between the planar aromatic surface and the nucleobases, supplemented by electrostatic interactions (if the anthraquinone bears cationic substituents, for instance amino side chains) and hydrogen bonding (from hydroxyl or carboxamide groups) [12,13,71]. The binding constant (*K*_a_) for simple anthraquinones ranges from 10^3^ to 10^5^ M^−1^, increasing to 10^6^–10^7^ M^−1^ for derivatives with extended conjugation or cationic side chains, such as mitoxantrone and amsacrine [12,13,72].

Intercalation induces structural distortion in the DNA helix, trapping topoisomerase–DNA cleavable complexes and preventing religation, which leads to DNA double-strand breaks [12,13,35,36,37]. This mechanism underlies the antitumor activity of the anthracycline antibiotics. DNA intercalation is, however, inherently nonspecific: anthraquinones bind to any accessible DNA sequence, causing genotoxicity in proliferating normal tissues such as bone marrow and intestinal crypts, contributing to myelosuppression and mucositis [60,73,74,75,76].

Peptidyl and hybrid anthraquinones have been designed to improve DNA-binding selectivity. Anthraquinone–amino acid conjugates, for example, exhibit preferential binding to G-quadruplex DNA structures (non-canonical four-stranded structures formed in guanine-rich sequences, prevalent at telomeres and oncogene promoters) over duplex DNA, with selectivity ratios exceeding 100:1 [77,78,79] (Figure 2). This selectivity arises from shape complementarity: the planar anthraquinone core stacks on the terminal G-quartet, while cationic amino acid side chains interact with the negatively charged phosphodiester loops [77,78,79]. Such compounds stabilize G-quadruplexes, inhibit telomerase, and suppress transcription of oncogenes including *c-MYC* and *BCL-2*, offering a more targeted mechanism than nonspecific intercalation [77,78,79].

### 2.4. Why the Scaffold Is a SAR Platform for Drug Design

The anthraquinone scaffold is unusually well suited as a platform for systematic SAR exploration and rational drug design for four reasons.

*Multiple, independently addressable substitution sites.* The eight peripheral positions (1–8) plus the quinone carbonyls (9, 10) provide at least ten sites for functionalization. Substituents at α-positions primarily modulate redox and chelation properties, while β-position substituents predominantly affect lipophilicity and pharmacokinetics [8,9,14,15]. This spatial orthogonality allows dissection of structure–activity contributions.

*Predictable electronic effects.* Substituent effects on quinone reduction potential follow well-established physical organic principles (Hammett correlations), enabling computational prediction of redox activity [8,9]. Similarly, chelation affinity can be estimated from hard-soft acid-base theory (HSAB) and ligand field stabilization energies [8,9]. This predictability reduces empiricism in lead optimization.

*Well-characterized mechanisms.* Unlike many natural product scaffolds whose biological targets remain obscure, anthraquinones engage defined molecular targets with established assays and crystal structures (topoisomerases, MMPs, DNA, mitochondrial respiratory complexes, kinases) [65,68,69,70,80,81,82]. This mechanistic clarity facilitates structure-based design.

*Clinical validation.* The therapeutic efficacy of doxorubicin, mitoxantrone, and amsacrine provides clinical proof-of-concept, de-risking the scaffold and justifying investment in derivative synthesis [1,31,60,83]. Furthermore, the known toxicities (cardiotoxicity, myelosuppression) are mechanism-based rather than idiosyncratic, implying that structural modifications that decouple activity from toxicity are, in principle, achievable [60,61,62].

Together, these features establish the anthraquinone core as a privileged scaffold for SAR-guided drug discovery. The following sections examine how specific substitution patterns within monomeric, dimeric, and hybrid structures affect the balance between redox cycling, chelation, DNA intercalation, and off-target toxicity, providing a framework for targeted drug design.

## 3. Structure–Activity Relationships of Monomeric Anthraquinones

### 3.1. The Significance of Hydroxyl Groups: Hydroxylation Versus Methoxylation

Hydroxyl (–OH) substitution is the single most important determinant of the biological profile of natural and synthetic anthraquinones, shaping redox potential, metal chelation, and molecular recognition alike [18,19,84]. What matters is not merely the number of hydroxyls but their placement: the distinction between α-positions (C-1, C-4, C-5, C-8) and β-positions (C-2, C-3, C-6, C-7) governs both the thermodynamic stability of the semiquinone radical and the acidity of the phenolic protons [8,9].

As described in Section 2, the strong intramolecular hydrogen bonding between α-hydroxyl groups and the adjacent C-9 or C-10 carbonyl lowers the hydroxyl pK_a_ to approximately 6.5–7.5. Consequently, this group undergoes partial ionization at physiological pH, while the resulting semiquinone radical is stabilized via PCET [8,9,53]. In antioxidant assays such as DPPH and ABTS, the number of free α-hydroxyl groups correlates positively with radical-scavenging capacity [9,10]. Purpurin (1,2,4-trihydroxy) and alizarin (1,2-dihydroxy) accordingly outperform emodin (1,3,8-trihydroxy), the decisive factor being the ortho-dihydroxyl motif that permits formation of a stable catechol-like radical [8,9,51,52,53] (Figure 3).

Methoxylation, a conversion of a hydroxyl to a methoxy group, functions as a structural switch in anthraquinone SAR. By removing the mobile hydrogen atom required for intramolecular hydrogen bonding and PCET, methoxylation reshapes the redox profile in a fundamental way. Its effects can be grouped under three headings.

*Shift in redox potential.* Methoxylated derivatives, physcion (the 6-methyl ether of emodin) being the canonical example, exhibit more negative reduction potentials (E_1_/_2_) than their hydroxylated parents [10,50]. Greater resistance to enzymatic one-electron reduction follows, along with diminished ROS generation and, frequently, lower acute cytotoxicity toward normal cells [54,85].

*Loss of chelation.* Methoxylation at C-1 or C-2 eliminates the bidentate coordination site on which metal binding depends [8,9]. Methoxylated anthraquinones consequently cannot inhibit metal-dependent enzymes such as prolyl-4-hydroxylase or the MMPs through the chelation mechanism [65,68].

*Altered lipophilicity and permeability.* Methoxy groups raise the octanol–water partition coefficient (LogP) of the scaffold appreciably. Passive diffusion across the plasma membrane may improve as a result, yet the concurrent loss of hydrogen-bond-donating capacity often weakens binding to DNA and to protein targets that require specific H-bond anchors [14,15,86] (Figure 3). Methyl substitution increases lipophilicity (logP) with minimal effect on hydrogen-bond donor capacity, thereby enhancing passive diffusion across lipid bilayers while retaining the free hydroxyl groups necessary for polar interactions with bacterial membrane components. Methoxy substitution also increases lipophilicity but eliminates the hydrogen-bond donor, which appears to be critical for the membrane-disrupting activity of anthraquinones against bacteria: the free hydroxyl is proposed to anchor the compound to the membrane surface via hydrogen bonds with phospholipid head groups, facilitating insertion of the planar core.

In toxicology, the hydroxylation-versus-methoxylation balance serves as a lever for modulating hepatotoxicity and nephrotoxicity. Emodin, a trihydroxylated molecule, is repeatedly implicated in dose-dependent liver injury mediated by bile acid dysregulation and mitochondrial oxidative stress [87,88,89]. Physcion, its 6-methoxy analogue, generally shows a more favourable toxicological profile in mice—an outcome attributed to reduced propensity for redox cycling together with altered metabolic routing [90,91].

### 3.2. Hybridization and Side-Chain Extension

Natural anthraquinones are, for the most part, simple hydroxylated or methoxylated monomers. Drug design frequently departs from this pattern, employing hybridization and side-chain extension to sharpen target affinity and selectivity [1,29,92] (Figure 4). Nowhere is this more evident than in the antitumor antibiotics, mitoxantrone and its modern analogues among them.

Introduction of aminoalkyl side chains (–NH–CH_2_–CH_2_–OH and related motifs) has proved the most successful extension strategy. Such cationic or polar chains serve several distinct SAR functions.

*Stabilization of DNA binding.* While the anthraquinone core intercalates between base pairs, extended side chains occupy the DNA minor or major groove, where they form additional hydrogen bonds and electrostatic contacts with the phosphate backbone [57,58,93]. This anchoring raises the binding constant (*K*_a_) and lengthens the residence time of the drug on DNA, a prerequisite for effective trapping of topoisomerase II–DNA cleavable complexes [35,36,37].

*Enzyme specificity.* Side chains can reach distal pockets within enzyme active sites. Among heteroarene-fused anthraquinones developed as Aurora B kinase inhibitors, the length and rigidity of the substituent at C-2 or C-3 determine selectivity over other kinase isoforms [81,82].

*Circumvention of resistance.* Hybridization with heterocyclic motifs—triazoles and furans, for instance—can alter recognition by efflux pumps such as P-glycoprotein. Anthra[2,3-b]furan-3-carboxamides retain potent activity against P-glycoprotein-overexpressing multidrug-resistant (MDR) cell lines, since their expanded planar surface and particular carboxamide side chains bypass active transport [34,36,72].

A more recent line of work within rational drug design concerns peptidyl–anthraquinone conjugates. Linking the core to cell-penetrating peptides (CPPs) or tumor-homing sequences such as RGD motifs has enabled targeted delivery to malignant cells, with a marked reduction in the systemic ROS-mediated toxicity that has long limited the classical anthracyclines [94,95].

### 3.3. Methoxy, Alkyl, and Related Substitutions

Alkyl (typically methyl) and methoxy groups are widely distributed among natural anthraquinones, with chrysophanol (1,8-dihydroxy-3-methyl) and physcion (1,8-dihydroxy-3-methyl-6-methoxy) serving as prominent examples [18,19]. These substituents are not incidental biosynthetic decoration; they provide essential tuning of the physicochemical platform. A methyl group at C-3, present in emodin, chrysophanol, and physcion alike, is the signature of the acetate–malonate biosynthetic pathway in plants [18]. From an SAR standpoint it contributes in three ways.

*Increased lipophilicity.* LogP rises without appreciable perturbation of the electronic properties of the quinone core, which aids cellular uptake [14,15].

*Steric modulation.* In interactions with enzymes such as pancreatic lipase or DPP-IV, a C-3 methyl group may supply favourable hydrophobic contacts or, depending on the geometry of the target pocket, introduce steric hindrance that prevents binding altogether [96,97].

*Metabolic protection.* Alkyl groups can block specific hydroxylation sites, thereby redirecting metabolism and potentially limiting the formation of reactive, toxic metabolites [87,88].

SAR studies employing fluorescence microscopy show that the ratio of hydroxyl to methoxy and alkyl substituents dictates where anthraquinones accumulate within the cell. Polyhydroxylated monomers such as alizarin tend to localize in the cytoplasm and lysosomes, consistent with their higher polarity and metal-binding affinity [8]. More lipophilic alkylated and methoxylated derivatives, chrysophanol among them, accumulate preferentially in the nucleus, which facilitates direct interaction with DNA [14,15,39,86].

Computational models based on DFT and three-dimensional quantitative SAR (3D-QSAR) have begun to systematize these observations. Total electronic energy and the HOMO–LUMO gap both prove strongly sensitive to the number of methoxy groups: as –OCH_3_ substitution increases, LUMO energy rises, and this rise correlates with a decrease in the first-electron reduction potential and a corresponding fall in the rate of ROS generation [8,9,16,80]. The practical value of such a framework lies in the rational attenuation of anthraquinone toxicity. Replacing α-hydroxyls with methoxy groups yields softer redox agents that retain DNA-binding capability while inflicting less oxidative damage on cardiomyocytes [60,61,62].

Overall, then, the biological activity of monomeric anthraquinones rests on a finely tuned balance between redox-active hydroxyl groups on the one hand and lipophilic, redox-attenuating methoxy and alkyl substituents on the other. Rational design exploits these relationships to widen the therapeutic index, moving the class from nonspecific natural toxins toward targeted, site-specific pharmaceutical agents (Table 2). These generalizations hold only within comparable structural series; position and neighboring-group effects can reverse a trend, as illustrated by the matched pairs discussed above.

## 4. Dimeric and Hybrid Anthraquinones: Structural Diversity and Expanded SAR Insights

### 4.1. Dimerization and Scaffold Multiplication

Dimeric anthraquinones form a structurally distinct class in which two anthraquinone cores are joined covalently through C–C, C–O, or methylene bridges [92,100,101]. Their pharmacology is not reducible to the sum of the constituent monomers. Hypericin (from *Hypericum perforatum*), sennosides A and B (from *Cassia* spp.), and the fungal metabolite skyrin all illustrate this point [20,21,102,103,104,105]. Three factors account for the divergence: the covalent linkage generates an extended conjugated π-system, it fixes the spatial presentation of functional groups, and it frequently permits bivalent binding modes that raise target affinity and specificity well beyond monomeric values.

#### 4.1.1. Structural Classification of Dimers

Dimeric anthraquinones are classified by the oxidation state of the linkage and by connectivity [100,101] (Figure 5).

*Dianthrones* comprise two anthrone units, the 9,10-reduced form of anthraquinone, joined by a central C–C bond, typically at C-10 or across C-9,10′. Sennosides A and B, homodimeric rhein derivatives, are glycosylated dianthrones that behave as prodrugs, undergoing bacterial cleavage in the colon to liberate the active aglycone rheinanthrone [102].

*Dianthraquinones* consist of two fully oxidized cores connected through C–C, C–O–C, or extended π-bridges. Naphthodianthrones hypericin and pseudohypericin are the pharmacologically most significant members; both adopt a perylenequinone architecture in which two emodin-like units are fused with additional five-membered rings [20,21].

*Heterodianthrones* are asymmetric dimers assembled from two different monomeric units—rhein combined with aloe-emodin, for example [100,101]. Such constructs can express mixed pharmacological character drawn from both parent scaffolds.

#### 4.1.2. Mechanistic Consequences of Dimerization

*Enhanced DNA intercalation and topoisomerase inhibition.* DNA intercalation and topoisomerase I, IIa, and IIb inhibition are primary strategies for numerous approved oncology therapeutics. Among the earliest identified topoisomerase inhibitors employed in cancer treatment are anthracyclines, which structurally derive from anthraquinones. This class includes clinically approved agents such as doxorubicin, epirubicin, valrubicin, daunorubicin, and idarubicin. Additionally, emodin—a natural anthraquinone sourced from fungi and plants—specifically targets DNA topoisomerase II. It disrupts ATP hydrolysis and induces double-strand DNA breaks by stabilizing the topoisomerase II–DNA cleavage complex [1,106,107].

The expanded planar surface of a dimer (for instance, the molecular dimensions of hypericin are approximately 14 Å × 8 Å) can engage two adjacent base-pair steps simultaneously or bis-intercalate, raising the DNA binding constant *K*_a_ by one to two orders of magnitude relative to monomers [23,24]. Hypericin binds duplex DNA with *K*_a_ ≈ 10^6^–10^7^ M^−1^, a range comparable to that of the classical anthracyclines [23]. High-affinity binding of this kind stabilizes topoisomerase I–DNA cleavable complexes far more effectively than monomeric emodin or chrysophanol, which contributes to hypericin cytotoxicity against glioma, leukaemia, and colorectal cancer cell lines [106,107,108,109,110]. At the same time, it should be noted that for fused naphthodianthrones such as hypericin the dominant, best-documented mechanism is photodynamic rather than intercalative: although hypericin binds nucleic acids and lipid environments with high apparent affinity, direct evidence for classical bis-intercalation or for topoisomerase-I trapping in the dark is limited. Its cytotoxicity against glioma, leukaemia and colorectal lines is therefore attributed primarily to light-triggered singlet-oxygen generation (Section, photodynamic activation). The bis-intercalative, high-affinity DNA-binding mode is better exemplified by synthetic bis-anthraquinone and anthracycline dimers designed for that purpose.

*Photodynamic activation.* Extended conjugation shifts the absorption maximum of hypericin into the visible range (λ_max_ ≈ 590 nm), so that activation can be achieved with red light, which penetrates tissue considerably more deeply than UV [23,24]. Photoexcitation is followed by intersystem crossing to a long-lived triplet state (^3^Hyp*), which transfers energy efficiently to molecular oxygen and generates singlet oxygen (^1^O_2_) with a quantum yield Φ_Δ_ ≈ 0.73 [23]. This mechanism has been carried into Phase II clinical trials for cutaneous T-cell lymphoma (mycosis fungoides), where objective response rates near 75% were achieved with minimal systemic toxicity [25,26,27,28].

The SAR governing photodynamic activity turns on two variables: the degree of π-conjugation and the positions of the hydroxyl groups. Chlorinated hypericin derivatives exhibit even higher triplet yields due to the heavy-atom effect, which enhances spin–orbit coupling and promotes intersystem crossing. However, excessive chlorination increases dark cytotoxicity, thereby reducing the light-dependent selectivity that constitutes the primary advantage of photodynamic therapy [111].

*Selective antibacterial and antifungal activity.* Dimeric anthraquinones bearing rare C–O–C ether linkages, isolated from fungal endophytes, show selective activity against viruses and Gram-positive bacteria, including methicillin-resistant *Staphylococcus aureus*, with minimum inhibitory concentration values of 2–8 μg/mL [112,113]. SAR analysis attributes this to a dual mechanism unavailable to monomers: the dimer architecture permits simultaneous interaction with the bacterial membrane and disruption of membrane-associated respiratory enzymes [112,114]. The rigidity of the dimeric scaffold appears to fix the hydroxyl groups at spacings well matched to the peptidoglycan layer and lipoteichoic acids of the Gram-positive cell wall [114,115].

*Enzyme inhibition through bivalent binding.* Dimerization can yield bivalent inhibitors capable of bridging spatially separated subsites within an enzyme. Hypericin, skyrin and rugulosin inhibit α-glucosidase with IC_50_ ≈ 10–20 μM (using p-nitrophenyl-α-D-glucopyranoside as substrate in phosphate buffer, pH 6.8, 37 °C), appreciably below values recorded for monomeric anthraquinones [116]. In a comparable vein, 1,1′,8,8′-tetrahydroxy dianthrone derivatives act as allosteric inhibitors of protein tyrosine phosphatase 1B, an enzyme implicated in insulin resistance and type 2 diabetes [117]. Here one anthraquinone core occupies the catalytic site while the second engages an allosteric pocket, locking the enzyme in an inactive conformation [117]. No monomeric anthraquinone can reproduce this binding mode, which points to a largely unexploited role for dimers in the design of non-competitive enzyme inhibitors.

### 4.2. Hybrid Anthraquinones

Hybrid anthraquinones—molecules in which the core is covalently fused or tethered to a second pharmacophore, whether heterocycle, peptide, chalcone, or β-lactam—constitute a frontier in rational polypharmacology [30,31,32,33,92,98] (Figure 6). Hybridization differs from simple substitution in an important respect: it introduces entirely new binding motifs, allowing a single molecule to engage several therapeutic targets or to exploit mechanistic synergy between its component parts.

#### 4.2.1. Heterocycle-Fused Hybrids

Fusing nitrogen- or sulfur-containing heterocycles (furans, thiophenes, triazoles, imidazoles) to the 2,3- or 3,4-positions of the core produces rigidified, extended aromatic systems with improved topoisomerase inhibition and kinase modulation [118,119,120].

*Anthra[2,3-b]furan derivatives.* Anthra[2,3-b]furan-3-carboxamides, in which a furan ring is fused at the 2,3-positions, have proved among the most productive hybrid platforms [34,35,36,37]. These compounds inhibit topoisomerase I with a potency one to two orders of magnitude greater than that of simple emodin [36,37]. Tuning lipophilicity through the carboxamide side chain at C-3 confers passive uptake rapid enough to outpace P-glycoprotein efflux, effectively circumventing multidrug resistance [72]. A further practical advantage distinguishes them from the classical anthracyclines, which require intravenous administration: anthra[2,3-b]furans retain significant activity in murine tumour models when given orally, an outcome attributed to greater metabolic stability combined with favourable membrane permeability [34].

*Azole-fused anthraquinones.* Incorporating triazoles, thiazoles, or selenazoles into the framework has produced compounds that target G-quadruplex DNA—non-canonical four-stranded structures that form in guanine-rich sequences at telomeres and oncogene promoters [36,37]. Triazole-fused anthraquinones carrying cationic side chains at C-4 and C-11 stabilize telomeric G-quadruplexes in preference to duplex DNA (selectivity ratio > 100:1), inhibit telomerase (IC_50_ ≈ 0.5–2 μM), and suppress transcription of the *c-MYC* and *BCL-2* oncogenes [13,37,79] (Figure 6A). Molecular recognition involves two complementary mechanisms: the triazole nitrogens act as hydrogen-bond acceptors that interact with the guanine quartets, whereas the planar surface of the anthraquinone core facilitates π–π stacking [78]. This binding mode is encoded structurally and cannot be reproduced by the anthraquinone scaffold alone.

#### 4.2.2. Peptidyl and Amino Acid Conjugates

Conjugation to amino acids offers a route to introduce chirality, improve aqueous solubility, and modulate cellular uptake [79,86,95,121]. Chiral amino-acid-based anthraquinones assembled through proline or glycine linkers show dose-dependent antiproliferative activity against pancreatic, breast, and colorectal cancer lines, with IC_50_ values spanning 5–50 μM according to the amino acid used and the geometry of the linker [86,121]. These conjugates additionally suppress cell migration and clonogenic capacity, which argues for effects extending beyond simple cytotoxicity possibly involving disruption of cytoskeletal dynamics or integrin signalling [86].

A promising targeting strategy involves the conjugation of the anthraquinone core to peptide ligands, e.g., gonadotropin-releasing hormone (GnRH) (Figure 6E). Because GnRH receptors are overexpressed on hormone-dependent prostate, breast, and ovarian tumors, these conjugates facilitate receptor-mediated endocytosis. This peptide-driven mechanism delivers the anthraquinone payload directly into the tumor cytoplasm, thereby enhancing selectivity and minimizing systemic toxicity [94].

#### 4.2.3. Chalcone and Coumarin Hybrids

Chalcones—α,β-unsaturated ketones bridging two aromatic rings (Figure 6B)—are privileged scaffolds in anticancer design, valued for their capacity to inhibit tubulin polymerization and modulate NF-κB signalling [122,123,124]. Anthraquinone–chalcone hybrids prepared by aldol condensation exhibit synergistic cytotoxicity [123,124]. Constructs combining an emodin core with a chalcone moiety, for instance, give IC_50_ values of 2–10 μM against HeLa and MCF-7 cells, inducing S- and G2/M-phase arrest together with caspase-dependent apoptosis [123]. Mechanistic investigations reveal that both pharmacophores contribute independently to the molecule’s activity; the chalcone component impairs microtubule assembly, while the anthraquinone domain mediates DNA intercalation and topoisomerase inhibition. Notably, the combined lethality exceeds the sum of their individual effects [123,124].

Anthraquinone–coumarin hybrids (Figure 6C) follow a similar logic, recruiting the antibacterial and tyrosinase-inhibiting properties of coumarins while retaining the ROS-generating and metal-chelating capacity of the anthraquinone core [125]. Molecular docking together with DFT calculations suggests simultaneous engagement of two sites within tyrosinase, the copper active site through anthraquinone chelation and the substrate-binding pocket through the coumarin, which is consistent with tyrosinase inhibition at IC_50_ ≈ 5–15 μM, markedly better than either scaffold achieves independently [125]. These in silico results propose, but do not experimentally demonstrate, a dual-site binding mode; direct enzymological confirmation is still required.

#### 4.2.4. β-Lactam–Anthraquinone Hybrids

Fusion of β-lactam rings (azetidin-2-ones, Figure 6D) to the anthraquinone core has produced dual-action antibacterials that attack both cell wall synthesis, by inhibiting penicillin-binding proteins, and respiratory metabolism, through quinone-mediated ROS generation [126,127,128]. These hybrids act across a broad spectrum encompassing Gram-positive and Gram-negative pathogens, including strains resistant to conventional β-lactam antibiotics [128]. Their activity is not confined to bacteria: antimalarial potency has also been documented, with IC_50_ values of 0.5–5 μM against chloroquine-resistant *Plasmodium falciparum* [127]. The antimalarial mechanism proposed on the basis of activity data and analogy and not directly demonstrated centres on interference with hemozoin formation in the parasite digestive vacuole, where the anthraquinone core intercalates into heme dimers and blocks polymerization, while the β-lactam moiety may additionally inhibit parasite proteases [127].

## 5. Absorption, Metabolism, Distribution, and Excretion (ADME) of Anthraquinones

### 5.1. Absorbtion

The oral absorption of anthraquinones is governed primarily by their physicochemical form—whether they are present as aglycones or O-glycosides—and by the composition of the intestinal microbiota. Free aglycones such as emodin, aloe-emodin, rhein, and physcion possess sufficient lipophilicity for passive transcellular diffusion across enterocytes, yet their poor aqueous solubility at physiological pH substantially limits the effective dissolved concentration available for absorption [14,15,18,19]. In contrast, anthraquinone glycosides (e.g., sennosides, cascarosides, aloin) are too hydrophilic for direct small-intestinal absorption and reach the large intestine largely intact, where they are hydrolysed by colonic β-glucosidases and reductases to release the corresponding aglycones and anthrone/anthranol intermediates [18,19,102]. This microbiome-dependent activation introduces marked interindividual variability in systemic exposure. Additionally, P-glycoprotein (P-gp/ABCB1)-mediated efflux at the apical membrane of enterocytes limits the net absorption of several anthraquinones and contributes to their low apparent oral bioavailability [8,12].

### 5.2. Distribution

Following absorption, anthraquinone aglycones are extensively bound to plasma proteins, primarily albumin, with free fractions typically below 10% [14,15]. Tissue distribution studies indicate preferential accumulation in the liver, kidney, and intestinal wall, consistent with high expression of relevant transporters (OATP1B1, OATP1B3) in these organs [14,15]. Penetration across the blood–brain barrier is negligible for glycosidic forms and limited even for lipophilic aglycones under physiological conditions, which has implications for both the central pharmacology and the neurotoxicity profile of this compound class.

### 5.3. Metabolism

Hepatic phase I metabolism of anthraquinone aglycones proceeds mainly via CYP1A2- and CYP3A4-catalysed hydroxylation; emodin, for example, yields ω-hydroxyemodin and 2-hydroxyemodin as primary oxidative metabolites [87,88]. Phase II conjugation—glucuronidation by UGT1A and UGT2B isoforms and sulfation by SULT1A1—substantially increases polarity and drives biliary and urinary excretion [88,89]. 3-O- and 8-O-glucuronides of emodin represent the predominant circulating species in vivo, and their relative abundance shifts with dose and species [63,88]. First-pass hepatic extraction is substantial for most monomeric anthraquinones, further compressing systemic exposure following oral dosing.

### 5.4. Excretion and Enterohepatic Recirculation

Excretion occurs predominantly via the biliary-fecal route for the parent aglycones, while conjugated metabolites are recovered in urine. Enterohepatic recirculation of glucuronide conjugates—involving biliary secretion, intestinal deconjugation, and reabsorption of the released aglycone—prolongs the effective half-life and sustains low plasma concentrations well beyond the absorption phase [18,19]. Reported elimination half-lives range from approximately 3–6 h for rhein to over 20 h for emodin under conditions of enterohepatic cycling, underscoring the risk of cumulative exposure with repeated dosing in nutritional supplements [63,88].

Taken together, the oral bioavailability of anthraquinone aglycones in humans is generally low (estimated 1–10% for emodin and rhein under fasted conditions), reflecting the combined constraints of limited dissolution, P-glicoprotein efflux, extensive first-pass metabolism, and rapid biliary clearance [14,15,88]. Formulation strategies—including cyclodextrin complexation, self-emulsifying drug delivery systems, and nanoparticulate carriers—have been shown to improve dissolution rate and permeability, thereby shifting the bioavailability window [44,45,46,47,48,49]. For nutritional supplementation contexts, where prolonged low-dose exposures are typical, the interplay between gut microbiota composition, hepatic metabolic capacity, and transporter expression represents a critical determinant of both efficacy and genotoxic risk, and should be considered when establishing acceptable daily intake levels.

## 6. Toxicity of Anthraquinones

Despite their potent biological activities, anthraquinones carry significant systemic toxicities that constrain their clinical use. Understanding the relationship between structure and toxicity is essential if the therapeutic window is to be widened by design. A recurring difficulty in the anthraquinone toxicology literature is that data derive from two very different sources: purified single compounds tested at defined doses, and complex botanical preparations (e.g., Polygonum multiflorum, Rheum, senna extracts) whose toxicity reflects the whole matrix—the relative content of free aglycones, glycosides and dianthrones, co-extracted constituents, batch variability, and the dose and duration of exposure. Effects observed for an extract cannot be attributed to a single anthraquinone without compositional and dose data, and conversely the toxicity of an isolated compound may be modified (enhanced or attenuated) within a botanical matrix. Throughout this section we therefore indicate, where the primary data allow, whether a finding refers to an isolated compound or to a preparation, and note the exposure conditions.

### 6.1. Hepatotoxicity

Hepatotoxicity is the adverse effect most frequently reported for anthraquinone-containing herbal preparations, especially those derived from *Polygonum multiflorum* and *Rheum* species [87,88,89,90]. Hepatocellular injury induced by anthraquinones is mediated by several distinct biochemical mechanisms. First, emodin and related rhein-type anthraquinones upregulate hepatic bile acid transporters, specifically the bile salt export pump (BSEP) and multidrug resistance-associated protein 2 (MRP2), while inhibiting conjugating enzymes such as sulfotransferase 2A1 (SULT2A1). This dual effect leads to the intracellular accumulation of unconjugated bile acids, subsequently inducing cholestasis, oxidative stress, and hepatocellular apoptosis [88,89]. Second, anthraquinones with low reduction potentials, such as emodin and aloe-emodin, undergo redox cycling within hepatocyte mitochondria. The resulting generation of ROS depletes intracellular glutathione and triggers mitochondrial membrane depolarization [87,91]. Hepatocytes, which combine high metabolic activity with a dense mitochondrial population, are especially vulnerable. Third, cytochrome P450-mediated oxidation can generate reactive electrophiles such as quinone methides that form covalent adducts with hepatic proteins and trigger immune-mediated liver injury [87,91].

Comparative data point to a clear structure–toxicity trend. In vitro hepatocyte assays combined with in silico reverse dosimetry indicate that rhein-type compounds (rhein, emodin, aloe-emodin) are considerably more hepatotoxic than alizarin-type compounds (alizarin, purpurin), with predicted human toxic doses differing by roughly an order of magnitude [88]. The concentration of dianthrones in the extracts shows a positive correlation with hepatotoxicity; however, traditional processing via steaming or stir-frying mitigates this risk by lowering dianthrone levels and enhancing overall safety [129,130].

### 6.2. Nephrotoxicity

Long-term use of anthraquinone-rich laxatives (e.g., senna, cascara) can induce renal complications, spanning acute tubular necrosis and chronic tubulointerstitial nephropathy [131,132]. Pathogenic pathways involve ROS-induced epithelial damage, altered renal hemodynamics via prostaglandin inhibition, and tissue fibrosis triggered by pseudomelanosis. Characteristically, anthraquinones exhibit a biphasic dose–response. While high doses induce nephrotoxicity, low doses protect against acute kidney injury by upregulating the Nrf2 pathway and suppressing IL-6 and TNF-α [133]. This dual effect confirms a narrow therapeutic index, requiring strict dosing strategies.

### 6.3. Genotoxicity

The DNA-intercalating and redox-active properties of anthraquinones also confer inherent genotoxic potential [85,134]. In mammalian cell cultures, aloe-emodin induces micronuclei, DNA strand breaks, and chromosomal aberrations. Furthermore, its positive response in the Ames assay confirms its capacity to induce genetic mutations [85]. Rhein, in contrast, is generally Ames-negative, which suggests that specific structural features—the C-3 carboxylic acid in this case—can modulate genotoxicity. Mechanistically, the damage arises both directly (ROS-mediated 8-oxoguanine formation and guanine adducts) and indirectly (topoisomerase poisoning and accumulation of cleavable complexes) [135,136].

### 6.4. Anthraquinones in Dietary Supplements: Standardization, Dosing, Metabolism and Regulation

Unlike pharmaceutical anthraquinones, botanical supplements (senna, aloe, cascara, rhubarb) are typically standardized only to total hydroxyanthracene derivatives, if at all, and vary in the ratio of free aglycones to glycosides and dianthrones—the very parameters that govern both laxative efficacy and hepatotoxic potential. Metabolically, glycosidic prodrugs (sennosides, barbaloin) are activated by colonic microbiota to rheinanthrone, so inter-individual differences in microbiota, transit time and co-medication (e.g., antibiotics) markedly affect exposure. Relevant interactions include potentiation of potassium loss with diuretics and corticosteroids and altered absorption of co-administered drugs. Regulatory attention has intensified: the European Food Safety Authority has concluded that hydroxyanthracene derivatives raise genotoxicity/carcinogenicity concerns, and several jurisdictions (the European Union Regulation (EU) 2021/468, the United States FDA Compliance Policy Guide, Sec. 490.200) now restrict permitted daily amounts and require warnings against prolonged unsupervised use [137,138]. These considerations reinforce the central argument of this review—that replacing poorly defined botanical mixtures with characterized single substances, dosed and standardized on SAR-informed criteria, is the rational route to safer products.

## 7. Strategies for Toxicity Mitigation

The toxicities detailed above are inherently linked to the same physicochemical properties that drive the biological activity of anthraquinones, namely redox cycling, DNA intercalation, and metal chelation. However, these adverse effects are not immutable; rational structural modifications and advanced formulation strategies can decouple therapeutic efficacy from toxicity. To achieve this, several established approaches have been developed.

### 7.1. Methoxylation and Glycosylation

Blocking free hydroxyl groups suppresses redox cycling and ROS generation. Glycosylated derivatives such as barbaloin and the sennosides act as prodrugs that intestinal bacteria cleave, achieving localized activation in the colon and minimizing systemic exposure [18,19,102].

### 7.2. Liposomal Encapsulation

Liposomal formulations of mitoxantrone and doxorubicin show improved pharmacokinetics, reduced cardiotoxicity, and greater tumor accumulation through the enhanced permeability and retention effect [44,45,46,47,48,49]. Recent Phase II data for liposomal mitoxantrone in relapsed T-cell lymphoma report a better therapeutic index than the free drug [48,49].

### 7.3. Targeted Delivery by Conjugation

Conjugating anthraquinones to tumor-homing peptides (such as RGD motifs or GnRH analogs) or monoclonal antibodies selectively concentrates the drug within malignant tissues, thereby minimizing toxicity in healthy organs [94,95]. Hybrid constructs bearing cell-penetrating peptides have shown 5- to 10-fold selectivity for cancer cells over normal fibroblasts in vitro [95].

### 7.4. Photodynamic Selectivity

For photosensitizing dimers such as hypericin, localizing light exposure to the tumor site via fiber-optic delivery or topical application confines cytotoxic ROS production to malignant tissues, thereby mitigating systemic toxicity [25,26,27,28].

## 8. Conclusions

Evidently, tailoring the substituent profiles of monomeric anthraquinones or developing dimeric and hybrid architectures allows for a significant tuning of their pharmacological properties. Rational structural modifications, combined with innovative nanoformulations (e.g., liposomes and nanoparticles), may improve the balance between therapeutic efficacy and toxicity, thereby improving the pharmacokinetic and bioavailability profiles of these candidates.

Translational medicine requires a clear differentiation between pharmaceutical-grade anthraquinones and those present in dietary supplements. Despite chemical similarities, these products are not equivalent in manufacturing quality, dosage precision, evidence-based efficacy, or toxicological evaluation. Because anthraquinones exhibit diverse and often contradictory pharmacological profiles, their multifunctionality is beneficial only within structured drug design; for unsupervised consumer use, it introduces unpredictable risks.

Simultaneous laxative, pro-oxidant, and cytotoxic actions from unstandardized botanical preparations drastically elevate the likelihood of off-target toxicity. While analytical measures like chemical profiling and contaminant screening are vital, they remain insufficient to fully ensure safety. Ultimately, implementing structure–activity relationship principles offers a reliable strategy to optimize both medicinal candidates and consumer supplement formulations.

## Figures and Tables

**Figure 1:**
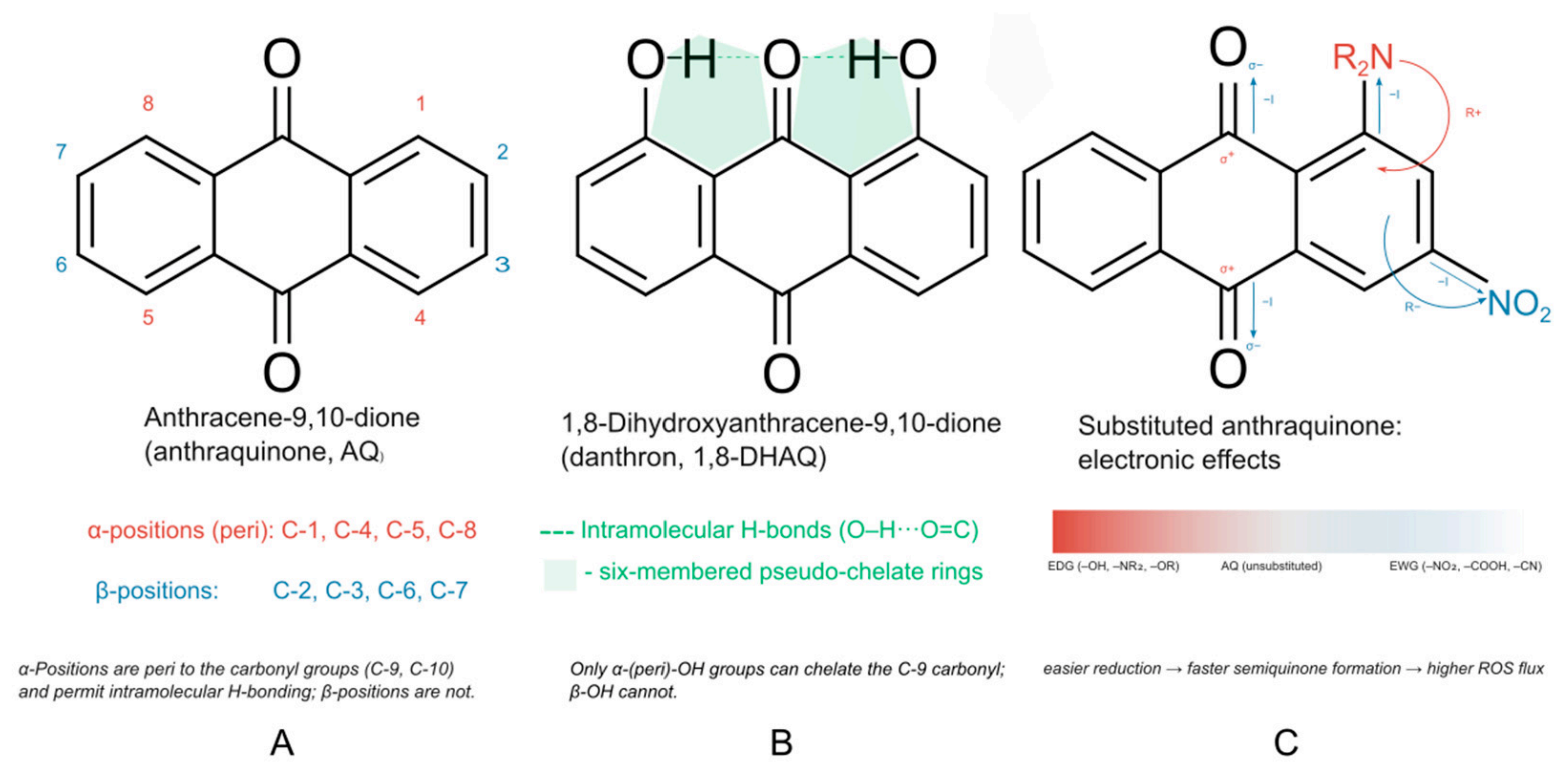
Structural and electronic basis of position-dependent reactivity in anthraquinones. (**A**) Numbering of the anthracene-9,10-dione (anthraquinone, AQ) core; α-(*peri*)-positions C-1, C-4, C-5, C-8 are shown in red, β-positions C-2, C-3, C-6, C-7 in blue. (**B**) Intramolecular hydrogen bonding in 1,8-dihydroxyanthracene-9,10-dione (danthron, 1,8-DHAQ): each α-OH group donates an O–H···O=C bond to the C-9 carbonyl, closing a six-membered pseudo-chelate ring (O, H, O, C-9, C-9a/C-8a, C-1/C-8); green dashed lines denote hydrogen bonds. β-OH groups cannot form such contacts. (**C**) Electronic effects of representative substituents on the AQ core: partial charges (δ^+^/δ^−^), resonance contributions (curved arrows, ±R) and inductive contributions (straight arrows along σ-bonds, −I). Throughout the figure, red denotes electron-donating/α-type character and blue denotes electron-withdrawing/β-type character.

**Figure 2:**
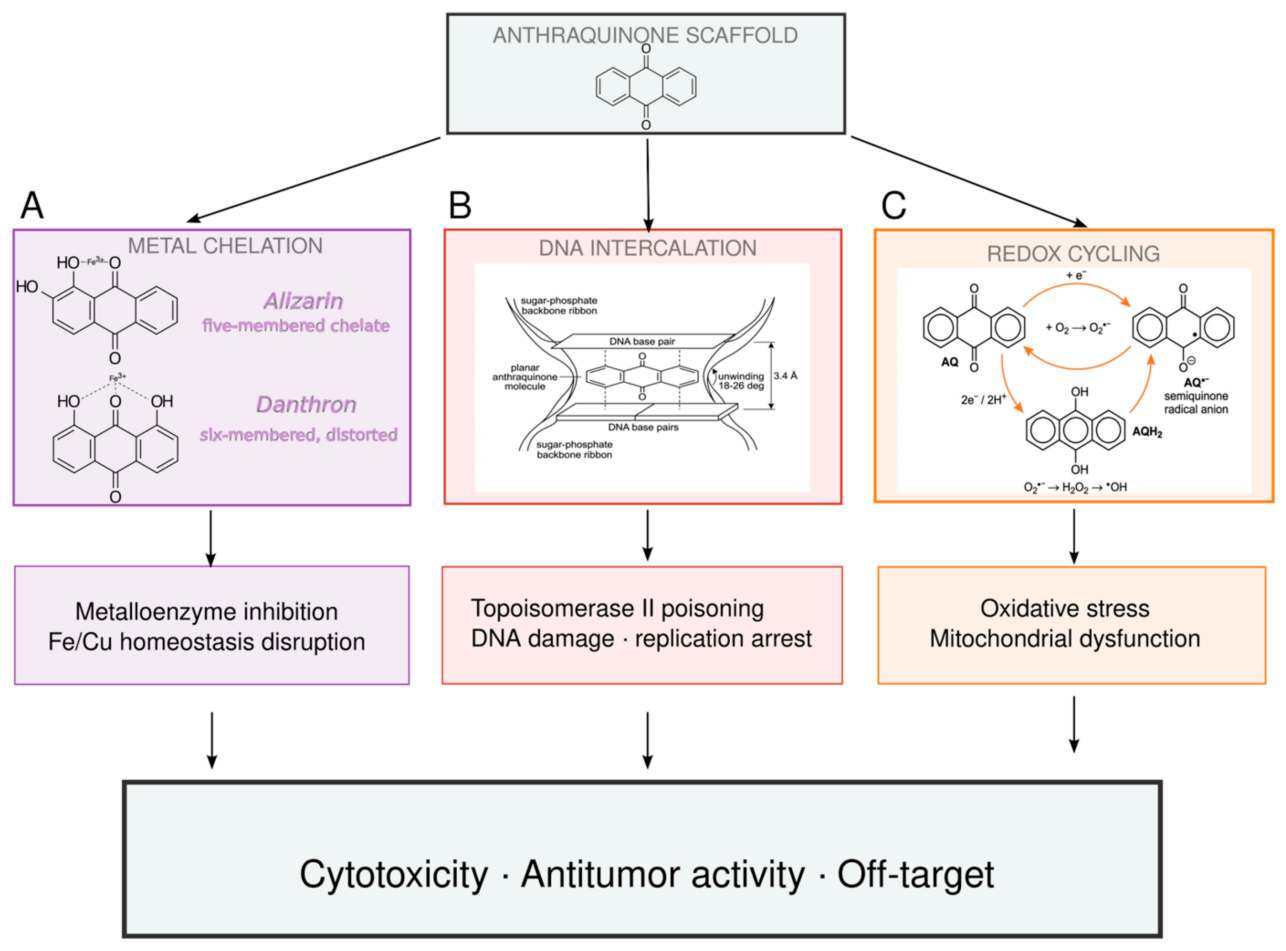
Three mechanisms converging on a single scaffold. The anthraquinone (AQ) core (centre) engages biological targets through three mechanistically distinct modes. (**A**) Metal chelation: 1,2-dihydroxy substitution forms a planar five-membered chelate with Fe^3+^, whereas 1,8-(*peri*)-dihydroxy substitution gives a six-membered chelate with distorted, non-planar geometry and correspondingly weaker binding; dashed purple lines denote coordination bonds. (**B**) DNA intercalation: the planar AQ core inserts between adjacent base pairs at a stacking distance of ≈3.4 Å, unwinding the duplex by 18–26°. (**C**) Redox cycling: one-electron reduction generates the semiquinone radical anion (AQ•^−^), which transfers the electron to molecular oxygen to yield superoxide (O_2_•^−^) and regenerate the parent quinone. Arrows below each panel indicate the principal biological consequences; all three converge on the cytotoxic and antitumor phenotype (bottom bar). Colour coding is specific to this figure: purple—metal coordination, red—DNA interaction, orange—redox processes.

**Figure 3:**
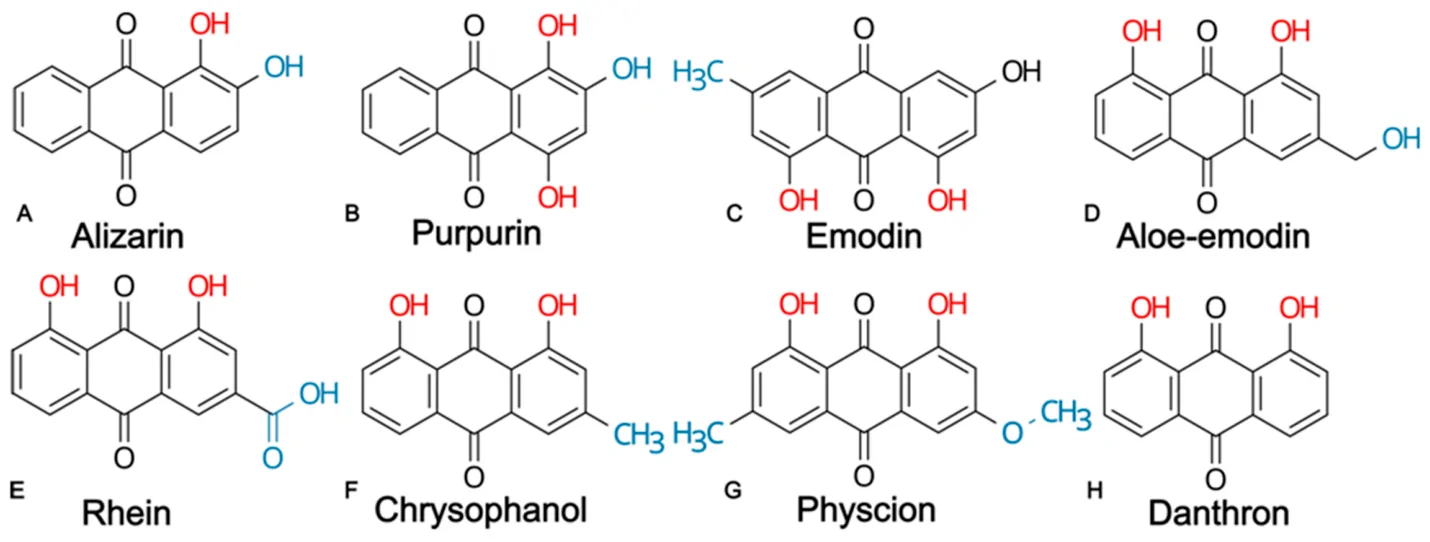
Representative monomeric natural anthraquinones. α-Hydroxyl groups (red; positions 1, 4, 5, 8) engage in intramolecular hydrogen bonding with the *peri*-carbonyl and enhance redox activity; β-hydroxyl groups (blue; positions 2, 3, 6, 7) lack this H-bond geometry and contribute differently to electronic and chelation properties. Color coding applies to both the oxygen atom and its bonds. The alizarin-type scaffold (**A**,**B**) bears a catechol motif enabling metal chelation; the emodin/rhein-type (**C**–**F**) distributes hydroxyls across both (**A**,**B**) rings. Physcion (**G**) illustrates how O-methylation at C-6 attenuates chelation and shifts lipophilicity relative to emodin (**C**). Danthron ((**H**), 1,8-dihydroxyanthraquinone) serves as a minimal reference scaffold lacking the C-3 substituent.

**Figure 4:**
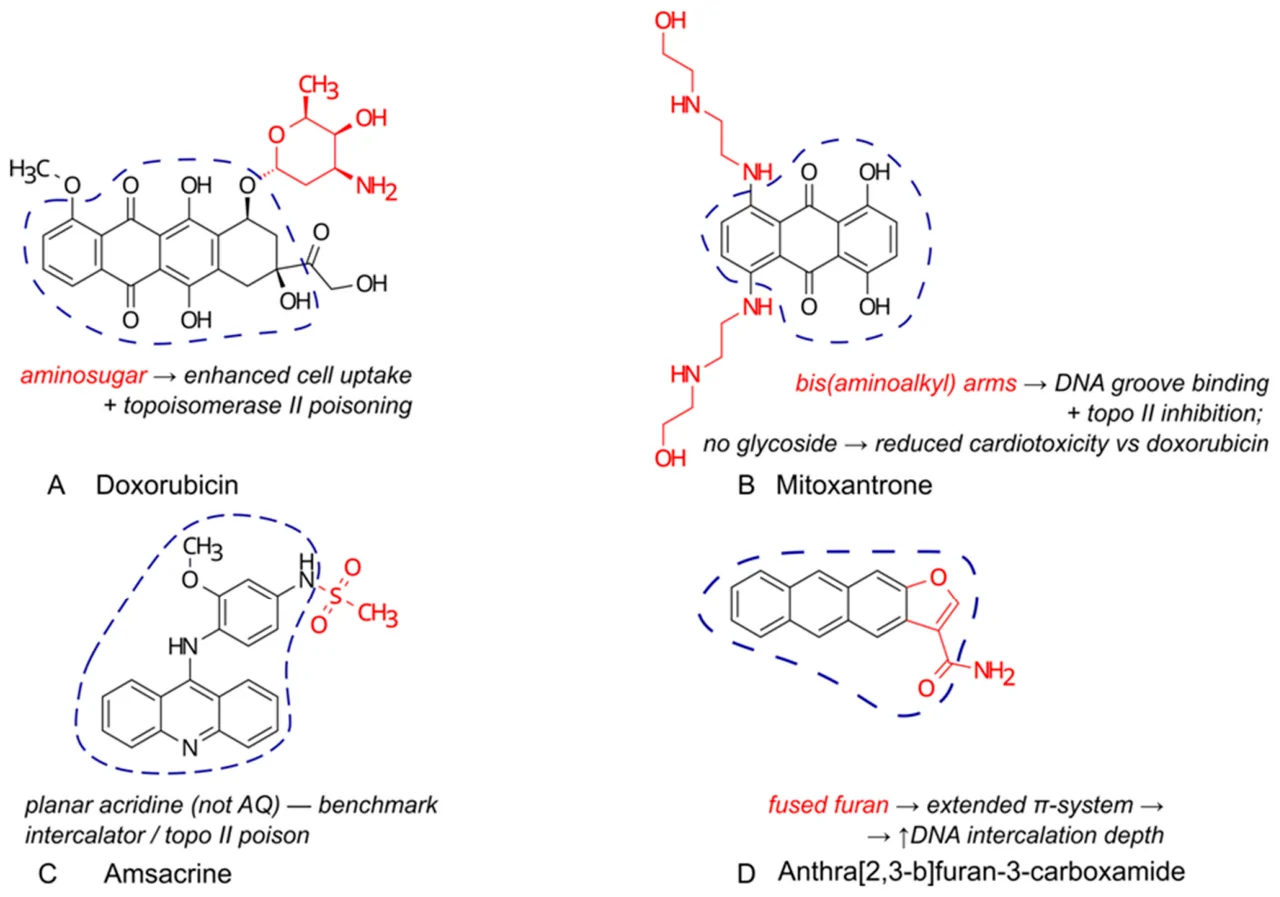
Synthetic monomeric anthraquinone-based agents and side-chain-extended scaffolds. The extended planar π-system (blue dashed outline) is conserved across all panels and mediates DNA intercalation. Unique pharmacophoric elements (red) determine target selectivity and ADMET profile: the daunosamine sugar in doxorubicin (**A**), bis(aminoalkyl) arms in mitoxantrone (**B**), the sulfonanilide in amsacrine ((**C**), acridine benchmark), and the fused furan–carboxamide in the anthra[2,3-b]furan series (**D**).

**Figure 5:**
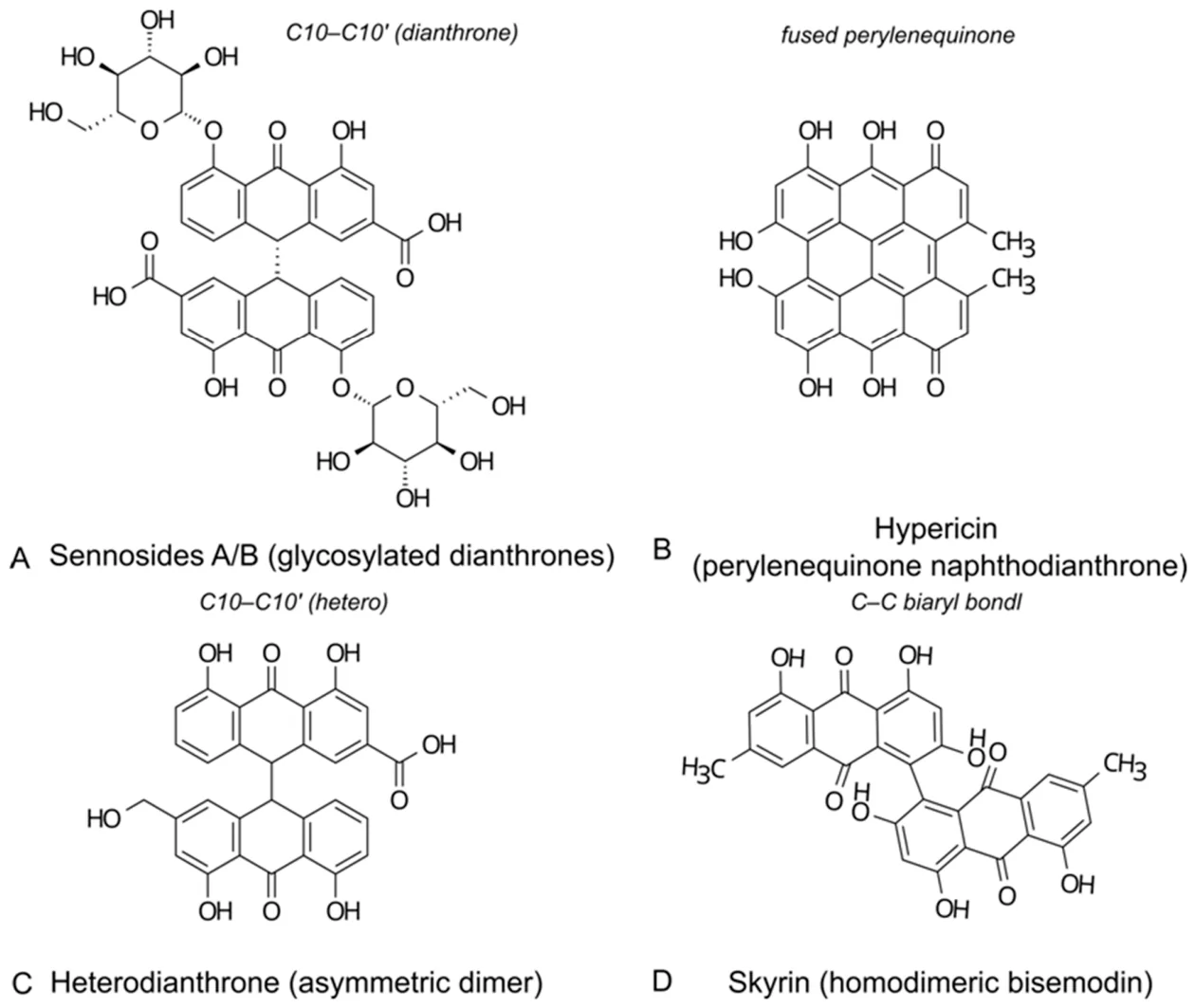
Structural classification of dimeric anthraquinones by linkage type. (**A**) Sennosides A/B: glycosylated C10–C10′ dianthrones that function as prodrugs, releasing the active laxative rheinanthrone upon colonic bacterial hydrolysis of both the glycosidic bonds and the dianthrone C–C bond. (**B**) Hypericin: a naphthodianthrone of the perylenequinone class, distinguished by a fully fused hexacyclic aromatic framework and seven hydroxyl groups; its predominant biological mechanism is photodynamic singlet-oxygen generation rather than DNA bis-intercalation. (**C**) Representative asymmetric heterodianthrone formed by C10–C10′ coupling of two structurally distinct monomers, illustrating the structural diversity accessible beyond homodimers. (**D**) Skyrin, a fungal homodimeric bisemodin linked via a C2–C2′ biaryl bond. Together, panels (**A**–**D**) demonstrate that “dimeric anthraquinone” encompasses at least four distinct structural classes differing in linkage chemistry, oxidation state, and pharmacological mechanism.

**Figure 6:**
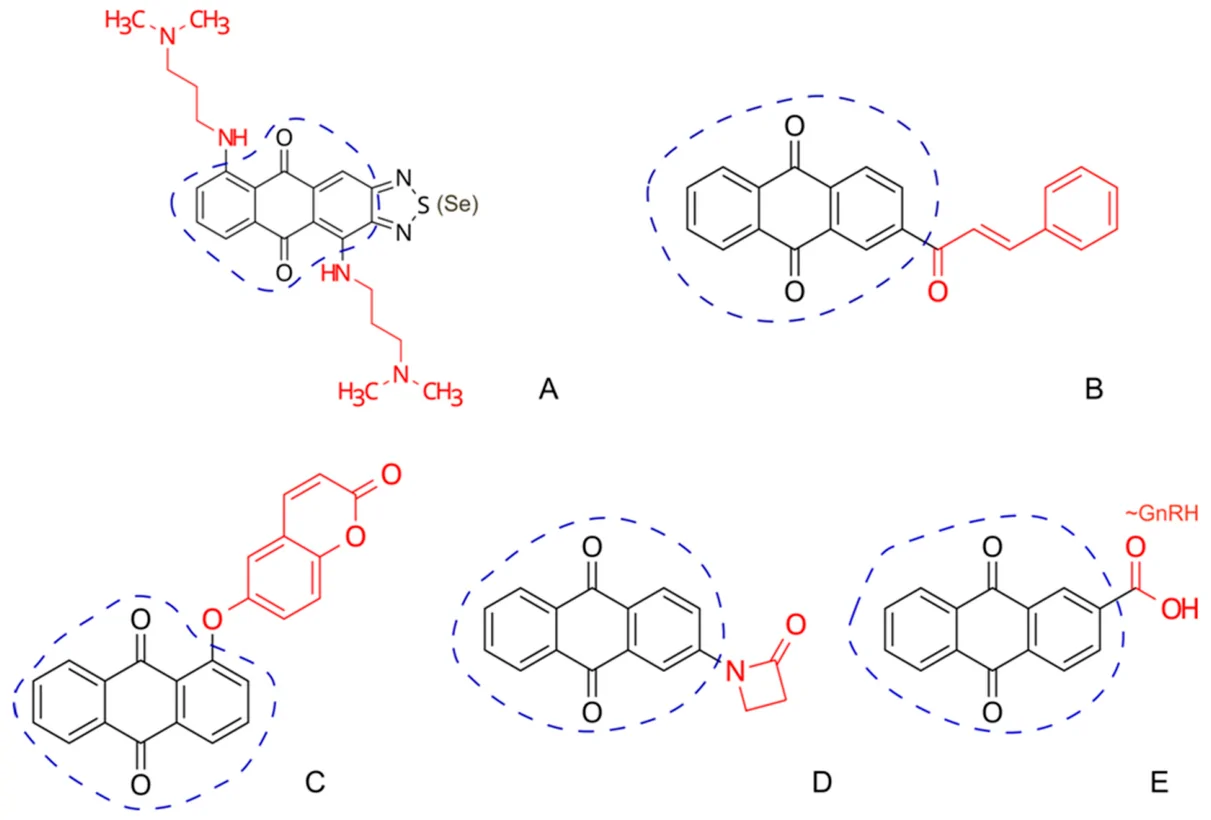
Hybrid anthraquinones obtained by covalent fusion or conjugation with a second pharmacophore, classified by linkage type. (**A**) 1,2,5-Thiadiazole (or selenadiazole/triazole) fused at C2–C3 extends the planar π-system and, combined with cationic aminoalkyl side chains at C-4/C-11, confers selective G-quadruplex stabilization. (**B**) Chalcone hybrids in which the anthraquinone acts as the aryl ketone component of an (*E*)-enone bridge; the resulting Michael acceptor provides a second electrophilic pharmacophore for cytotoxicity. (**C**) Coumarin–anthraquinone conjugates linked via an O-ether bond combine DNA intercalation with the photosensitizing singlet-oxygen capacity of the coumarin lactone. (**D**) *N*-Aryl β-lactam hybrids merge the four-membered lactam antibacterial pharmacophore with the anthraquinone intercalating scaffold into a single bifunctional agent. (**E**) Amino-acid and peptide (GnRH) conjugates formed through a C-2 carboxamide linkage; overexpression of GnRH receptors in tumor cells provides selectivity for targeted delivery. The conserved anthraquinone π-system (blue dashed outline) mediates DNA association in all panels; the appended pharmacophore (red) defines secondary target engagement, ADMET profile, and delivery strategy.

**Table 1:** Parameters of antioxidant activity of anthraquinones.

Anthraquinone	OH-Positions	C-3 Subst.	DPPH Scavenging Activity (IC_50_)	Redox Potential (E_1/2_ or Ep, vs. SCE)	LPO Inhibition, %	Ref.
**Purpurin**	1,2,4-triOH	H	≈15	−0.45…−0.50	85–90	[9,51]
**Alizarin**	1,2-diOH	H	12–25	≈−0.55	≈93	[51,52]
**Emodin**	1,3,8-triOH	CH_3_	35–64	≈−0.65	≈36	[8,51,53]
**Aloe-emodin**	1,8-diOH	CH_2_OH	>100	≈−0.72	≈78	[51]
**Rhein**	1,8-diOH	COOH	>150	≈−0.70	≈71	[51]
**Chrysophanol**	1,8-diOH	CH_3_	>200	≈−0.78	n.d.	[39,51,54]

**Note:** Electrochemical potentials (**E_1/2_**) or first reduction peak (**Ep**) are reported relative to SCE. Data originally measured against an Ag/AgCl reference electrode were converted to the SCE scale using the standard equation *E*vs. SCE = *E*vs. Ag/AgCl −0.044 V. Experimental conditions: glassy carbon working electrode, aprotic solvent (MeCN or DMF), 0.1 M Bu_4_ NPF_6_ as supporting electrolyte, scan rate 100 mV/s, temperature 25 °C. Caution: values in this table are compiled from independent studies employing different assays, solvents, reference electrodes and concentration ranges. DPPH IC_50_ values in particular depend strongly on assay time, DPPH: antioxidant ratio and solvent, and reduction potentials depend on solvent and electrolyte. The data should therefore be read as indicating qualitative trends within, not rigorous quantitative comparisons across, the cited studies. DPPH, 2,2-diphenyl-1-picrylhydrazyl; LPO, lipid peroxidation; n.d., not determined; SCE saturated calomel electrode.

**Table 2:** SAR of monomeric anthraquinones.

Functional Group	Antioxidant Activity	Antitumor Activity	Antibacterial Activity	Enzyme Inhibitory Activity	Ref.
Hydroxyl (–OH)	Enhances (highly active via radical scavenging)	Enhances (stabilizes DNA binding and triggers ROS)	Enhances (increases cell membrane permeability)	Enhances (provides hydrogen bonding anchors)	[83,98]
Methoxy (–OCH_3_)	Decreases (blocks radical-scavenging phenol)	Modulates (tunes cytotoxicity and reduces toxicity)	Variable (lowers efficacy due to lower polarity)	Modulates (can fit hydrophobic pockets or block binding)	[93,98]
Methyl (–CH_3_)	Negligible effect (electron-donating only)	Modulates (increases lipophilicity for cell entry)	Enhances (aids in penetrating bacterial lipids)	Modulates (alters affinity to specific active sites)	[83,98]
Amino (–NH_2_)	Negligible effect	Strongly Enhances (dramatically boosts DNA intercalation)	Enhances (cationic amino groups damage bacterial walls)	Strongly enhances (highly active against kinases/topoisomerases)	[79,99]
Glycosidic	Strongly decreases (eliminates free active phenolic –OH)	Modulates (lowers acute toxicity, alters cell-uptake pathways)	Decreases (bulky sugars block cell-wall passage)	Decreases (sterically blocks the enzyme’s active site)	[16,83]

Note: The trends summarized here are context-dependent: the effect of a given group varies with its ring position (α vs. β), the surrounding substitution pattern, and the biological target. Entries should be read together with the matched examples given in the text (e.g., emodin vs. physcion for –OH vs. –OCH_3_; chrysophanol vs. its glycoside for aglycone vs. glycoside) rather than as universal rules.

## Data Availability

No new data were created or analyzed in this study. Data sharing is not applicable to this article.

## Abbreviations

The following abbreviations are used in this manuscript:

ADMET	Absorption, Distribution, Metabolism, Excretion, and Toxicity
AQ	Anthraquinone
DFT	Density functional theory
DPPH	2,2-Diphenyl-1-picrylhydrazyl
GnRH	Gonadotropin-releasing hormone
HOMO	Highest occupied molecular orbital
IC_50_	Half maximal effective concentration
LUMO	Lowest unoccupied molecular orbital
MMP	Matrix metalloproteinase
MRSA	Methicillin-resistant Staphylococcus aureus
NADPH	Nicotinamide adenine dinucleotide phosphate
PCET	Proton-coupled electron transfer
ROS	Reactive oxygen species
SAR	Structure–activity relationship
SCE	Saturated calomel electrode
